# The levels of serine proteases in colon tissue interstitial fluid and serum serve as an indicator of colorectal cancer progression

**DOI:** 10.18632/oncotarget.8693

**Published:** 2016-04-11

**Authors:** Yingying Xie, Lechuang Chen, Xiaolei Lv, Guixue Hou, Yang Wang, Cuicui Jiang, Hongxia Zhu, Ningzhi Xu, Lin Wu, Xiaomin Lou, Siqi Liu

**Affiliations:** ^1^ CAS Key Laboratory of Genome Sciences and Information, China Gastrointestinal Cancer Research Center, Beijing Institute of Genomics, Chinese Academy of Sciences, Beijing, 100101, China; ^2^ University of Chinese Academy of Sciences, Beijing, 100049, China; ^3^ Laboratory of Cell and Molecular Biology and State Key Laboratory of Molecular Oncology, Cancer Hospital, Chinese Academy of Medical Sciences and Peking Union Medical College, Beijing, 100021, China; ^4^ Beijing Protein Innovation, Beijing, 101318, China; ^5^ Proteomics Division, BGI-Shenzhen, Shenzhen, Guangdong, 518083, China

**Keywords:** tissue interstitial fluid, serum, colorectal cancer, biomarker, serine proteases

## Abstract

The proteins in tissue interstitial fluids (TIFs) can spread into the blood and have been proposed as an ideal material to find blood biomarkers. The colon TIFs were collected from 8-, 13-, 18-, and 22-week Apc^Min/+^, a typical mouse model of colorectal cancer (CRC), and wild-type mice. iTRAQ-based quantification proteomics was conducted to survey the TIF proteins whose abundance appeared to depend on tumor progression. A total of 46 proteins that exhibited consecutive changes in abundance were identified, including six serine proteases, chymotrypsin-like elastase 1 (CELA1), chymotrypsin-like elastase 2A (CEL2A), chymopasin, chymotrypsinogen B (CTRB1), trypsin 2 (TRY2), and trypsin 4 (TRY4). The observed increases in the abundance of serine proteases were supported in another quantitative evaluation of the individual colon TIFs using a multiple reaction monitor (MRM) assay. Importantly, the increases in the abundance of serine proteases were also verified in the corresponding sera. The quantitative verification of the serine proteases was further extended to the clinical sera, revealing significantly higher levels of CELA1, CEL2A, CTRL/chymopasin, and TRY2 in CRC patients. The receiver operating characteristic analysis illustrated that the combination of CELA1 and CTRL reached the best diagnostic performance, with 90.0% sensitivity and 80.0% specificity. Thus, the quantitative target analysis demonstrated that some serine proteases are indicative of CRC progression.

## INTRODUCTION

Colorectal cancer (CRC) is one of the most dangerous cancers and characterized by a high incidence and mortality [1]. Despite dramatic improvements in the five-year survival rate of CRC patients diagnosed at the early stage, most patients are diagnosed too late to receive effective medical treatment due to a lack of sensitive diagnostic tools for asymptomatic individuals. CRC is currently diagnosed using three main methods: colonoscopy, fecal occult blood testing (FOBT), and biomarker detection. As a gold standard screening method, colonoscopy can detect subtle abnormal changes in the morphology of the intestinal epithelial layer and greatly improve the CRC diagnosis rate to over 50.0% [2–4]. However, it is not sufficiently sensitive to detect the morphological changes of early CRC, and it poses risks due to the invasiveness of the procedure. FOBT is an easily employed diagnostic method reported to reduce CRC mortality by approximately 20% [5], but its sensitivity and specificity for CRC, especially for early CRC, are poor because early precancerous lesions produce less occult blood in stool, whereas some non-cancerous intestinal diseases cause serious hemafecia [6]. At present, CRC diagnosis based on protein biomarkers in the serum has become an attractive area of study because cancer-related biomarkers are specific and blood-based assays are easily manipulated. However, a highly sensitive and specific clinically accepted biomarker for CRC is lacking. For instance, carcinoembryonic antigen (CEA) and carbohydrate antigen 19-9 (CA 19-9) are commonly used to diagnose CRC, but their sensitivities, 30-40% for CEA and 14.1% for CA 19-9, are poor [7–9]. Moreover, technical challenges are inevitable, such as those associated with globally surveying CRC-related proteins in the serum and the efficient verification of candidate biomarkers.

Diagnosis based on a blood test has been acknowledged as the ultimate goal of biomarker investigation. However, discovering biomarkers in the serum is difficult. First, highly abundant proteins, such as albumin and transferrin, account for approximately 99% of the total serum proteins and significantly interfere with biomarker screening, irrespective of the approach employed for biomarker exploration. Although these proteins are often depleted during serum preparation, this approach may also inadvertently deplete serum proteins of low abundance. Second, the serum contains the circulating representation of many organs and tissues, indicating physiological and pathological changes in the body. Because a representative protein signal from one target tissue is expected to be dramatically diluted when it is secreted into blood, these serum proteins require additional enrichment that enhances the sensitivity of protein extraction. However, methods to enrich poorly defined target proteins remain a challenge. In recent decades, tissue interstitial fluid (TIF) has been identified as a good material that circumvents the two disadvantages mentioned above and provides a promising avenue for biomarker discovery. TIF contains the proteins mainly secreted by cells in the tissue microenvironment and contains few highly abundant proteins. Moreover, because TIF is in close proximity to the cancer tissue source, the concentrations of proteins related to the pathological changes within it are reasoned to be higher than those of proteins in the serum. According to Ahn's estimation, the concentration of a potential biomarker in the local tumor microenvironment may be 1000-1500 times higher than that in blood [10]. The proteins in TIF drain from the lymph and then spread into the blood [11]. Therefore, cancer-related proteins in TIF are natural serum biomarker candidates, especially during the discovery phase. For instance, the ovarian cancer biomarkers stress-induced phosphoprotein 1 and peroxiredoxin 1 as well as the CRC biomarkers leucine-rich alpha-2-glycoprotein 1 and tubulin beta-5 chain were first identified in the corresponding TIFs and then further verified in the sera of ovarian and CRC cancer patients [12–14]. However, methods to identify proteins related to the early tumor in the TIF remain a challenge. TIF is generally obtained from surgical samples. Once a patient undergoes surgery, the tumor is assumed to be morphologically detectable in the patient. Thus, the tissues excised by surgery may provide information on the advanced stage but not the early stage of the cancer. A dilemma is that on one hand the technical advantages of TIF should be utilized, while on the other hand human tissues with different stages of tumor progression could be collected for the TIF preparation. We propose a well-selected CRC animal model as a good solution to this problem.

The C57BL/6J-Apc^Min/+^ (Apc^Min/+^) mouse model is a clinically relevant model of early CRC that features a nonsense mutation at codon 850 of the adenomatous polyposis coli (*APC)* gene, which results in a truncated protein and predisposes the mouse to both small and large intestinal adenomas [15]. The pathological phenotype of the Apc^Min/+^ mouse is similar to that of human familial adenomatous polyposis and over 60% of sporadic CRC cases [16], and the profiles of gene expression and metabolomics in Apc^Min/+^ mice shared dearrangement of metabolic pathways with human ones [17]. Moreover, most tumors in Apc^Min/+^ mice are benign adenomas that neither aggressively invade nor metastasize [18, 19]. Therefore, this model is widely accepted to mimic the early stage of human colon carcinogenesis. Tumor progression is commonly observed to be a multistep process due to a series of cumulative genetic alterations that affect the homeostasis of biologic functions. Given the difficulty in collecting the human samples at different progression stages, animal models offer an opportunity to dynamically monitor the molecular events that accompany tumor growth. Apc^Min/+^ mouse model is postulated as an ideal TIF material for dynamically surveying the CRC-progression-dependent proteins. Importantly, the proteomics analysis would not stop at the discovery phase in the mouse model, and we also extended the verification of CRC-related proteins in human serum.

In this study, we analyzed the dynamic changes in the proteome of colon TIFs from Apc^Min/+^ and C57BL/6J mice during CRC development using iTRAQ quantification, and we verified the CRC-related proteins in mouse TIFs and sera using targeted multiple reaction monitor (MRM) quantification. Furthermore, these proteins were verified using MRM in human sera and a tissue microarray (TMA) analysis in human tissues. The iTRAQ data revealed that the levels of some colon TIF proteins in Apc^Min/+^ mice dynamically changed in response to CRC development. Of these proteins, the levels of six serine proteases, chymotrypsin-like elastase 1 (CELA1), chymotrypsin-like elastase 2A (CEL2A), chymopasin, chymotrypsinogen B (CTRB1), trypsin 2 (TRY2), and trypsin 4 (TRY4), increased in the Apc^Min/+^ TIFs, and the MRM results confirmed these changes in both the mouse TIFs and sera. Based on the MRM and TMA assay, we concluded that the levels of CELA1 and CTRL in colon tissues and sera were significantly higher in CRC patients than in healthy individuals. Thus, some serine proteases in the serum are likely indicative of CRC progression.

## RESULTS

### Monitoring CRC development in Apc^Min/+^ mice

**Figure 1:**
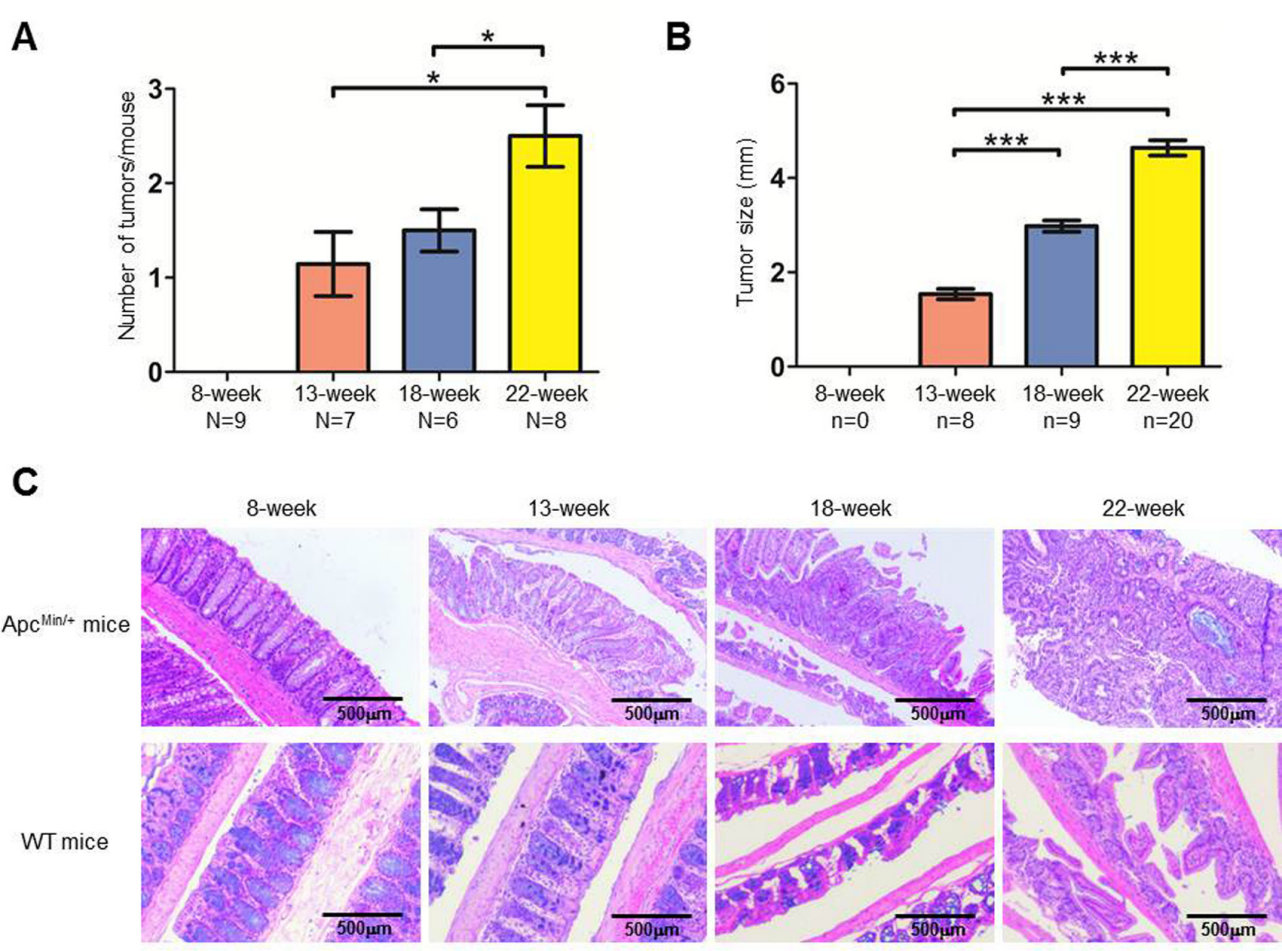
Characteristics of colorectal tumor development in the Apc^Min/+^ mouse model **A-B.** The average number of tumors **(A)** and tumor size **(B)** at each time point. The number of mice (N) and tumors (n) are indicated. Bars, mean with SE. **C.** H&E staining of colon tissues from Apc^Min/+^ and WT mice.

Genotype-verified Apc^Min/+^ and C57BL/6J (WT) mice were fed a high-fat diet starting at 3 weeks old. Most Apc^Min/+^ mice developed colon tumors by the age of 9 weeks and rarely survived longer than 23 weeks. Apc^Min/+^ and WT mice of different ages (8, 13, 18, and 22 weeks) were sacrificed, and the number and sizes of tumors in the colon as well as the colonic morphology were examined. Tumors were not detected in the colons of WT mice, and both the number and sizes of colon tumors in Apc^Min/+^ mice were directly correlated with the age of the mouse (Figure 1A, 1B and Supplementary Table 1). The morphological changes clearly indicated tumor development in Apc^Min/+^ mice: at 8 weeks, the colon glandular tubes remained an orderly arrangement, and slight lymphocyte infiltration was apparent; at 13 weeks, in addition to obvious lymphocyte infiltration, aberrant crypt foci and an elevated nuclear/cytoplasmic ratio could be detected; at 18 and 22 weeks, the nuclear/cytoplasmic ratio was significantly elevated, and obvious architectural atypia, such as disorderly colon glandular tubes, could be observed (Figure 1C). Therefore, all data demonstrated that the samples obtained from 8-, 13-, 18- and 22-week-old Apc^Min/+^ mice depicted CRC development.

### iTRAQ-based quantification proteomics of CRC-progression-dependent TIF proteins

**Figure 2:**
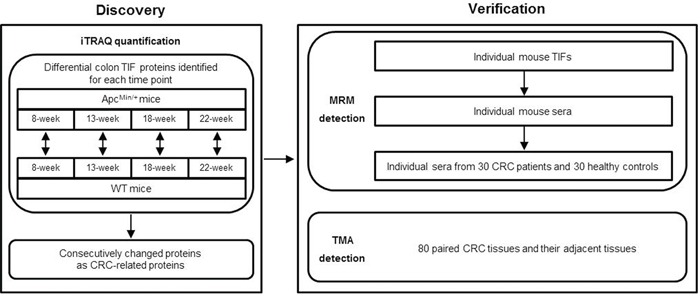
The experimental workflow for CRC biomarker discovery and verification

Our experimental strategy is illustrated in Figure 2. The colon TIF proteins generated from 8-, 13-, 18- and 22-week old Apc^Min/+^ and age-matched WT mice were analyzed by iTRAQ quantification, and proteins that were differentially expressed between Apc^Min/+^ and WT mice were identified for each time point to exclude differences due to mouse age. Differential proteins that exhibited consecutive changes were defined as CRC-related proteins and further verified in mouse and human samples by MRM and TMA analyses.

Equal amounts of TIF proteins from three mice at each time point were pooled for the iTRAQ analysis. Western blotting demonstrated a lack of organelle contamination in the TIF sample, which was then qualified for further proteomic analyses (Supplementary Figure 1). Technical duplicates were performed for LC-MS/MS. In the protein identification, the overlapped ratios of unique peptides and proteins between technical duplicates were 80.7% and 88.6%, respectively (Supplementary Figure 2A). In the protein quantification, the Pearson correlation analysis of the normalized tag intensities of spectra revealed a high reproducibility, with correlation coefficients exceeding 0.93 between technical duplicates at all four time points (Supplementary Figure 2B). Thus, the spectra from technical duplicates were combined for further analysis. A total of 6028 unique peptides corresponding to 1174 proteins (≤1% FDR and ≥2 unique peptides) were identified (Supplementary Table 2), and almost all of them (99%) were identified with iTRAQ tags.

**Figure 3:**
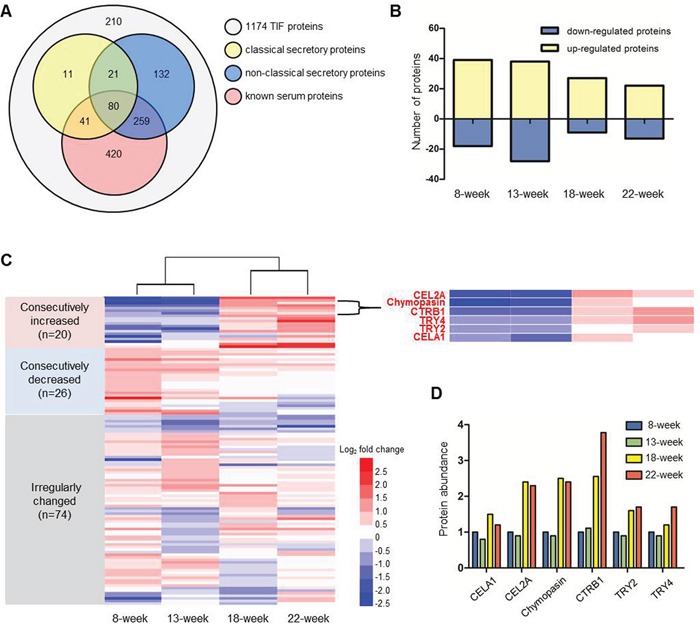
iTRAQ proteomic analysis of TIF proteins **A.** Secretory characteristics of 1174 colon TIF proteins. Classical and non-classical secretory proteins were predicted by SignalP 4.1 and SecretomeP 2.0. Known mouse serum proteins were reported in published papers [22, 23]. **B.** The number of differential proteins defined by comparing Apc^Min/+^ mice with age-matched WT mice. **C.** Heat map illustrating the change patterns (consecutive increase, consecutive decrease and irregular change) of 120 differential proteins. The enlarged region shows the consecutive increases in six serine proteases. **D.** The changes in the relative levels of the six serine proteases in Apc^Min/+^ mice. The level of each protein at 8 weeks was used as the reference.

Because TIF proteins are mainly released by cells in the tissue, they should possess secretory properties. Therefore, two software programs, SignalP 4.1 [20] and SecretomeP 2.0 [21], and an in-house “mouse serum protein database” generated from mouse serum proteins identified in the literature [22, 23] were adopted to predict the secretion potentials of the identified TIF proteins. As shown in Figure 3A, approximately 46.3% (544/1174) of TIF proteins were predicted to be secretory proteins. In addition, approximately 68.1% (800/1174) of the identified TIF proteins had been identified in the serum (Figure 3A), indicating that TIF proteins may be promising serum biomarkers.

Stringent criteria were established to identify TIF proteins that are differentially expressed between Apc^Min/+^ and WT mice: a differentially expressed protein was defined by a ≥1.5- or ≤0.67-fold change in abundance at a *p*≤0.05. A total of 120 differentially expressed proteins were identified; specifically, 39, 38, 27, and 22 proteins were up-regulated, and 18, 28, 9, and 13 proteins were down-regulated proteins at four time points (Figure 3B and Supplementary Table 2). Figure 3C shows the relative changes in protein expression during tumor progression by comparing Apc^Min/+^ to age-matched WT mice. These changes were more dramatic from week 13 to week 18 than from week 8 to week 13 and from week 18 to week 22. The 120 differentially expressed proteins could be divided into three groups: 20 consecutively increased, 26 consecutively decreased, and 74 irregularly changed. Among the 20 consecutively increased proteins, we found six proteins, CELA1, CEL2A, chymopasin, CTRB1, TRY2, and TRY4, that belonged to the same serine protease family and exhibited the exactly same change patterns (Figure 3C and 3D), suggesting that the changes in the protein levels of this family tightly correlated with CRC progression. In addition, the software prediction and literature search identified all six proteins as secretory proteins that could be detected in the mouse serum [22]. All evidence indicated that these six proteins may be promising serum biomarkers of CRC.

### Verification of the six serine proteases in the mouse colon TIFs and sera

**Figure 4:**
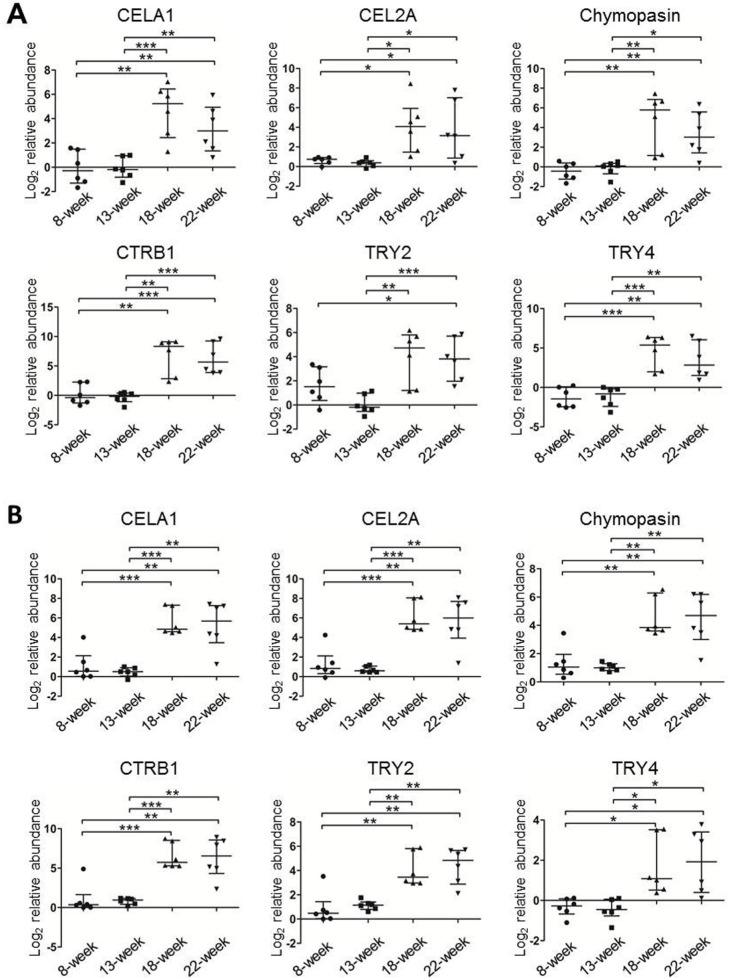
The relative protein levels of the six serine proteases in individual TIF (A) and serum (B) samples from Apc^Min/+^ mice (n=6/time point) measured using MRM The protein peak areas of one 13-week-old mouse and one 8-week-old mouse were used as the references for the TIF and serum samples, respectively. * *p*≤0.05, ** *p*≤0.01, *** *p*≤0.001 (Mann-Whitney U test). Bars, median with interquartile range.

To confirm the changes in the levels of the six serine proteases identified by iTRAQ, we employed MRM to detect these proteins in six newly prepared individual TIF samples of Apc^Min/+^ mice at each time point. We first used pooled TIF samples to establish the MRM method. Only CELA1 and CEL2A were detected among the peptides prepared from whole TIF proteins (data not shown). Because the molecular weights of the six proteins are all approximately 25-30 kDa, in-gel digestion of target sized proteins separated by SDS-PAGE was applied to increase the detection signal intensity of target peptides by reducing the sample complexity. Consequently, all the six proteins were readily detected by TripleTOF 5600 MS. One or two high-confidence unique peptides with four or five transitions were selected for each target protein (Supplementary Table 3), and corresponding synthetic peptides were used to verify the retention time and extracted ions. The transition orders and retention time of all 11 peptides were the same as and similar to those of their synthetic peptides, respectively (Supplementary Figure 3), indicating that these peptides were qualified for MRM quantification. We then measured individual samples from Apc^Min/+^ mice. Based on a Mann-Whitney U test, the levels of all six proteins were low at 8 and 13 weeks and significantly increased at 18 and 22 weeks (Figure 4A). Moreover, MRM was also performed in pooled TIF samples from three WT mice at each time point, and the results revealed that the levels of the six proteins were higher at 8 weeks and gradually decreased as the mice aged (Supplementary Figure 4A). The MRM results of TIFs from Apc^Min/+^ and WT mice were in agreement with the iTRAQ data, which further proved that the increases in the levels of the six proteins were related to tumor progression rather than mouse age.

Using the same MRM approach, we next measured the levels of these six proteins in six individual serum samples from Apc^Min/+^ mice as well as in a pooled serum sample from three WT mice at each time point. Similar to the levels of the six serine proteases in TIFs, the abundance in sera of Apc^Min/+^ mice was significantly higher at 18 and 22 weeks than at 8 and 13 weeks (Figure 4B). In WT mice, only CELA1, TRY2, and TRY4 could be detected and exhibited irregular change patterns (Supplementary Figure 4B), and the average intensities of the detected peptides for the three proteins were at least 30 times lower than those in 18- and 22-week Apc^Min/+^ mice. Therefore, all MRM results demonstrated that the serum abundance of the six proteins, CELA1, CEL2A, chymopasin, CTRB1, TRY2, and TRY4, reflected tumor development and served as CRC serum biomarkers in this mouse model.

### Verification of the six serine proteases in human sera and colorectal tissues

**Figure 5:**
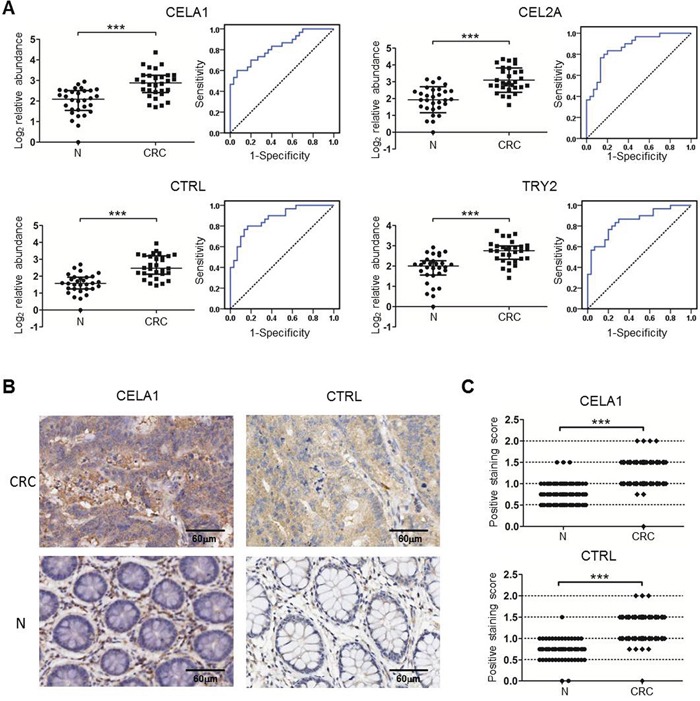
Verification of the serine proteases using MRM and TMA in individual human samples **A.** The relative protein levels and ROC curves of the four serine proteases in human sera based on an MRM analysis. The protein peak area of one healthy control was used as the reference. * *p*≤0.05, ** *p*≤0.01, *** *p*≤0.001 (Mann-Whitney U test). Bars, median with interquartile range. The solid lines in the ROC curves represent the plot of 1-specificity versus sensitivity. **B-C.** Representative images **(B)** and statistical analysis **(C)** of TMA detection of CELA1 and CTRL in 80 pairs of human CRC and their adjacent tissues (N). *** *p*≤0.001 (Paired-Samples T test).

To test whether the six proteins could be useful as serum biomarkers of human CRC, sera from 30 CRC patients and 30 healthy controls were collected (Table 1), and the levels of the six proteins were measured using the MRM approach. Because of differences in the sequences of the six serine proteases between humans and mice, the transitions used in mouse samples could not be used in humans. Using both identification via TripleTOF 6600 MS for 25-30 kDa proteins in pooled serum samples and the prediction of appropriate peptides via the skyline software, one or two high-confidence unique peptides in human serum corresponding to five transitions for CELA1, CEL2A, CTRL (chymotrypsin-like, human homologue of mouse chymopasin), and TRY2 each were selected for the relative quantification analysis (Supplementary Table 4). These peptides were also confirmed using chemically synthesized peptides (Supplementary Figure 5). We then measured individual serum samples and found that the levels of CELA1, CEL2A, CTRL, and TRY2 were significantly higher in the CRC sera than in the healthy controls (Figure 5A), as observed in the sera of mice. The receiver operating characteristic (ROC) curves illustrated that the serum levels of CELA1, CEL2A, CTRL, and TRY2 robustly distinguished CRC patients from healthy controls, with areas under the curve (AUC) of 0.83, 0.88, 0.87, and 0.85, respectively (Figure 5A and Table 2). These results indicated that CELA1, CEL2A, CTRL, and TRY2 may serve as human CRC serum biomarkers. A combination analysis identified CELA1 and CTRL as the best combination to distinguish CRC from healthy controls, with 90.0% sensitivity and 80.0% specificity (Table 2).

**Table 1:** Clinical characteristics of CRC patients and healthy controls for MRM detection

Characteristics	Colon cancer group (N=30) n (%)	Control group (N=30) n (%)
Gender		
Male	18 (60.0%)	17 (56.7%)
Female	12 (40.0%)	13 (43.3%)
Age		
31-40	5 (16.7%)	5 (16.7%)
41-50	9 (30.0%)	9 (30.0%)
51-60	9 (30.0%)	10 (33.3%)
61-70	7 (23.3%)	6 (20.0%)
Tumor size (cm)		
2-4	9 (30.0%)	
4-6	13 (43.3%)	
6-8	8 (26.7%)	
Depth of invasion		
T1	0	
T2	4 (13.3%)	
T3	15 (50.0%)	
T4	11 (36.7%)	
Lymph node involvement		
N0	15 (50.0%)	
N1	7 (23.3%)	
N2	8 (26.7%)	
Distant metastasis		
M0	29 (96.7%)	
M1	1 (3.3%)	

**Table 2:** ROC analysis and diagnostic performance of the four serine proteases based on serum MRM detection

Proteins	AUC	Sensitivity	Specificity	*p* value
CELA1	0.83 (95%CI, 0.72-0.93)	60.0%	93.3%	<0.001
CEL2A	0.87 (95%CI, 0.78-0.96)	83.3%	80.0%	<0.001
CTRL	0.87 (95%CI, 0.79-0.96)	76.7%	86.7%	<0.001
TRY2	0.85 (95%CI, 0.75-0.94)	80.0%	76.7%	<0.001
CELA1&CEL2A	0.88 (95%CI, 0.80-0.97)	96.7%	66.7%	<0.001
CELA1&CTRL	0.90 (95%CI, 0.83-0.98)	90.0%	80.0%	<0.001
CELA1&TRY2	0.90 (95%CI, 0.83-0.98)	86.7%	80.0%	<0.001
CEL2A&CTRL	0.90 (95%CI, 0.83-0.98)	86.7%	80.0%	<0.001
CEL2A&TRY2	0.90 (95%CI, 0.83-0.98)	86.7%	80.0%	<0.001
CTRL&TRY2	0.89 (95%CI, 0.82-0.97)	83.3%	83.3%	<0.001
CELA1&CEL2A&CTRL	0.90 (95%CI, 0.83-0.98)	90.0%	80.0%	<0.001
CELA1&CEL2A&TRY2	0.89 (95%CI, 0.82-0.97)	73.3%	90.0%	<0.001
CELA1&CTRL&TRY2	0.89 (95%CI, 0.82-0.97)	83.3%	80.0%	<0.001
CEL2A&CTRL&TRY2	0.90 (95%CI, 0.83-0.97)	86.7%	80.0%	<0.001
CELA1&CEL2A&CTRL&TRY2	0.90 (95%CI, 0.83-0.98)	86.7%	83.3%	<0.001

Because high concentrations of CELA1 and CTRL were verified in the sera of CRC patients, the colon tissues of CRC patients should also express high levels of CELA1 and CTRL. The levels of these proteins were evaluated in TMAs containing 80 pairs of CRC tissues and corresponding normal tissues (Table 3). This evaluation detected CELA1 and CTRL primarily in tumor cells, although expression was detected in normal epithelial cells. Specifically, the levels of CELA1 and CTRL were significantly higher in CRC tissues than in normal tissues (*p*<0.001) (Figure 5B and 5C). Moreover, in tumor tissues, CELA1 and CTRL were detected in both tumor and stromal cells, and the levels of the two proteases were significantly increased in the tumor cells not in stromal cells. However, CELA1 and CTRL expression did not correlate with clinical TNM stage, despite significant differences in CELA1 expression between T2 and T3, T3, and T4. Nevertheless, the changes in the mean staining intensity for the four stages were irregular (T1=1.5, T2=1.1, T3=1.4, and T4=1.2). Taken together, the data from MRM and TMA confirmed that the levels of these proteins were higher in both sera and tissues of CRC patients, indicating their potential as serum biomarkers for human CRC.

**Table 3:** Clinical characteristics of CRC patients for TMA detection

Characteristics	Colon cancer group (N=80) n (%)
Gender	
Male	47 (58.8%)
Female	32 (40.0%)
Unknown	1 (1.2%)
Age	
21-40	3 (3.8%)
41-60	20 (25.0%)
61-80	50 (62.5%)
>80	5 (6.2%)
Unknown	2 (2.5%)
Tumor size (cm)	
2-4	28 (35.0%)
4-6	36 (45.0%)
6-8	11 (13.8%)
8-10	4 (5.0%)
>10	1 (1.2%)
Depth of invasion	
T1	2 (2.5%)
T2	12 (15.0%)
T3	39 (48.8%)
T4	27 (33.7%)
Lymph node involvement	
N0	38 (47.5%)
N1	28 (35.0%)
N2	14 (17.5%)
Distant metastasis	
M0	68 (85.0%)
M1	12 (15.0%)

## DISCUSSION

Proteomic approaches have been widely used in biomarker discovery study, however few biomarkers discovered are applied in the clinical diagnosis. The problems are not so much with the technology but in the study design [24]. To discover the CRC-related biomarker candidates, we carefully designed four time points covering the whole tumor progression in Apc^Min/+^ mice and used age-matched WT mice as controls to exclude age-dependent changes. The screen of biomarker candidates were mainly based on change patterns with tumor progression and only the consecutively increased proteins were further verified in clinical samples.

Our findings provide two fundamental aspects of TIF proteomes. First, we found 46 TIF proteins that exhibited typical progression-dependent patterns of expression. Among these proteins, ribosomal protein (RP) S3, L4 and L14 were found up-regulated in colonic tumors of 68-day Apc^Min/+^ mice compared with normal epithelium from age-matched WT mice using ^15^N metabolic labeling and LC-MALDI-TOF/TOF-MS [25]. We also identified three RPs that significantly increased in TIFs from Apc^Min/+^ mice at weeks 8 and 13 compared with age-matched WT mice. However, these differences were negligible at weeks 18 and 22. Our study demonstrates that dynamic TIF proteomics can provide valuable information to effectively define CRC-related biomarker candidates. Second, many of the CRC-related TIF proteins may serve as serum biomarkers for CRC. Although the verification experiments in this study only focused on serine proteases in the colorectal TIF, we are confident that these candidates may be secreted into the serum. Specifically, a bioinformatics analysis predicted 58.7% (27/46) of CRC-related TIF proteins to be secretory proteins. Moreover, several experimental findings support our discovery. For example, the levels of S100A8 and S100A9 were significantly increased in the TIF at weeks 18 and 22, which corroborated the findings of Kim *et al.*: the levels of these two proteins were higher in the plasma of CRC and colorectal adenoma patients, and the areas under ROC curves for the two proteins were superior to that of CEA [26]. The levels of two TIF proteins, fibrinogen beta and gamma chain, were higher at 22 weeks than during the previous stages. Similar results were reported by Tang *et al*., who noted that the elevated plasma fibrinogen levels in CRC were associated with advanced tumor stage, venous invasion, and postoperative distant metastasis [27].

Based on a quantitative integrated analysis, six members of the serine protease family, CELA1, CEL2A, chymopasin, CTRB1, TRY2, and TRY4, were confirmed to be CRC-related proteins in the TIFs and sera of mice. Furthermore, CELA1, CEL2A, CTRL, and TRY2 were proven to be CRC biomarkers in human serum. Abnormal, often enhanced, expression and activity of serine proteases are widely reported in tumors [28, 29], and the proteases are involved in many physiological processes, including digestion, blood coagulation, fibrinolysis, the immune response, and signal transduction [30]. Because proteases possess cleavage activity to digest proteins, especially for matrix proteins, increased protease activity should be closely correlated with accelerated tissue protein digestion. In the tumor microenvironment, the cleavage of matrix proteins is likely to lead to the invasion and metastasis of cancer cells. Accordingly, the levels of proteases are increased in many solid tumors, including CRC [28, 31, 32]. For instance, Soreide *et al.* reported that trypsin activated a number of MMPs, which then helped to induce the progression, invasion, and metastasis of CRC [33]. Noteworthy, the activity of serine proteases on non-matrix subunits, such as chemokines, adhension molecules, growth factors, and apoptosis mediators, is essential for the critical cellular responses required for tumor progression [34, 35]. It should be noted that, in addition to taking functions based on enzyme activity, some serine proteases modulate the functions of cell surface receptors, such as proteinase activated receptors (PARs) and integrins [36]. For example, Ducroc *et al.* observed that human CRC cells produced and secreted trypsin at concentrations compatible with activation of PAR-2 to promote the survival and proliferation of the cancer cells, which suggested an autocrine/paracrine regulation of human colonic tumors by trypsin [37].

Our study provides two novel aspects of CRC-related serine proteases. First, our data clearly demonstrate that increases in the levels of several serine proteases in the TIFs and sera of Apc^Min/+^ mice occur during tumor growth. Therefore, these proteases may be markers of early CRC in blood. Second, previous studies of CRC-related serine proteases have primarily focused on members of the trypsin family, whereas we detected higher levels of two trypsins (TRY2 and TRY4) and four chymotrypsin or chymotrypsin-like proteases (CELA1, CEL2A, chymopasin, and CTRB1) in the TIFs and sera of Apc^Min/+^mice. Although trypsin and chymotrypsin belong to the serine protease family and share some substrates, the two kinds of proteases exhibit many differences in biochemical characteristics and physiological function and may perform very different roles in the tumor microenvironment. Lokhov *et al.* concluded that the trypsin digestion of the surface proteins on cancer cells generated essential antigens to induce an immune-mediated anti-tumor effect, which may help to develop an anti-tumor vaccine [38]. Moreover, Kondakova *et al.* revealed increases in the chymotrypsin-like activity of proteasomes in several cancer tissues compared with their adjacent tissues [39]. Chymotrypsin-like proteasome activation might play an important role in cancer pathogenesis. As regards whether there are special regulations to the chymotrypsin-related gene expression in colorectal mucosa cells or some matrix proteins in colorectal tissues favorable for chymotrypsin-like digestion, our results indeed establish a solid base for further functional investigation.

## MATERIALS AND METHODS

### Human sera collection

Human sera were collected from the Cancer Hospital of the Chinese Academy of Medical Sciences (CAMS), including serum samples from 30 CRC patients and 30 healthy controls (Table 1). None of the patients had taken any medication prior to surgery or venous blood draw. All experimental procedures described herein were reviewed and approved by the Institutional Review Board of the Cancer Hospital of the CAMS. Informed consent was obtained from all participants.

### Mouse breeding

All procedures used in this study were approved by the Institutional Animal Care and Use Committee of the Beijing Institute of Genomics at the Chinese Academy of Sciences. Male Apc^Min/+^ mice were purchased from Jackson Laboratory (Bar Harbor, ME) and bred with female WT mice from the CAMS. The mice were housed at room temperature under controlled humidity and light conditions with 12/12 light/dark cycle and received chow and tap water *ad libitum*. Three-week old pups were genotyped by PCR with the primers 5′-TTCCACTTTGGCATAAGGC-3′ and 5′-TTCTGAGAAAGACAGAAGTTA-3′ (Sangon Biotech, Shanghai, China) to identify Apc^Min/+^ mice. Both male Apc^Min/+^ and WT mice began to receive a high-fat diet providing 60 kcal% fat (Biotech-HD, Co. Ltd, Beijing, China) starting at age 3 weeks and were sacrificed at age 8, 13, 18, or 22 weeks. The colon tissues of Apc^Min/+^ and WT mice were sectioned and H&E stained for histological examination.

### Preparation of TIFs and sera

Blood was collected from individual mice via the retro-orbital sinus, and the serum was isolated via centrifugation at 1,500 g for 10 min after incubation at room temperature for 1 h. The colon tissue from each mouse was opened longitudinally and washed with PBS to remove the stool. After a quick measurement of the number and sizes of adenomas, the entire colon was dissected into 1-3 mm^3^ pieces and carefully rinsed 3 times with PBS. The dissected tissues were incubated with 200 μL PBS containing protease inhibitor cocktail (Calbiochem/Merck Millipore, Darmstadt, Germany) in a humidified incubator for 1 h at 37°C. The TIF protein specimens were collected via sequential centrifugation at 1,000 g for 5 min to remove cells, followed 5,000 g for 10 min and 12,000 g for 10 min at 4°C to remove debris. Both the serum and TIF samples were stored at −80°C.

### iTRAQ labeling of tryptic peptides derived from TIF proteins

Equal amounts of individual TIF proteins from three Apc^Min/+^ or WT mice at the same time point were pooled. One hundred micrograms of mixed proteins were reduced using 5 mM tris-(2-carboxyethyl) phosphine for 1 h at 60°C and alkylated by 10 mM methanethiosulfonate for 10 min at room temperature, followed by a 16 h trypsin digestion at 37°C. The tryptic peptides from 8, 13, 18, and 22-week-old Apc^Min/+^ mice were labeled with the 8-plex iTRAQ reagents 113, 114, 115, and 116, and the corresponding peptides from WT mice were labeled with 117, 118, 119, and 121, respectively, according to the manufacturer's instructions. The labeling reactions were carried out for 2 h; the labeled peptides were then mixed, and the reaction solvents were removed by a speed-vacuum.

### Peptide fractionation with Reverse phase (RP) chromatography

The mixed iTRAQ-labeled peptides were dissolved in buffer A (20 mM ammonium formate, pH=10), loaded onto a RP column (250×4.6 mm, 5 μm, 100 Å aperture, Luna C18, Phenomenex, Torrance, CA) in a Prominence HPLC system (Shimadzu, Kyoto, Japan). The flow rate was 1 ml/min, and the peptides were eluted by a step linear elution program: 0-20 min equilibration in 100% buffer A, 20-21 min fast elution with 0-12% buffer B (20 mM ammonium formate and 80% acetonitrile, pH=10), 21-56 min linear elution from with 56% buffer B, 56-61 min washing elution with 56-100% buffer B and 61-67 min elution with 100% buffer B. The peptides were monitored at 214 nm and collected in one tube/min during the elution period. The collected fractions were then lyophilized, dissolved in 0.1% formic acid and further pooled into 15 fractions to average the protein contents.

### Protein identification by LC-MS/MS analysis

The final 15 fractions were loaded in duplicate onto a nano-RP column (100×75 mm, 5 μm, 100 Å aperture, C18, Agela Technologies, Tianjin, China) mounted in a HPLC system (ultimate 3000, Dionex, Sunnyvale, CA) and eluted for 40 min at 400 nL/min with an acetonitrile gradient ranging from 5-30% containing 0.1% formic acid. The eluates were directly entered into an Q-Exactive MS (Thermo Fisher Scientific, Bremen, Germany) set to positive ion mode and data-dependent collection, with full MS scans ranging from 350-2000 m/z at a resolution of 70,000; MS/MS scans were conducted at a resolution of 175,000 and higher collision energy dissociation.

### iTRAQ data processing

The raw MS/MS data were converted into MGF format by Proteome Discoverer 1.3 (Thermo Scientific), and the exported MGF files were further compared with the IPI mouse v3.87 database (59534 entires) using Mascot 2.3 (Matrix Science) with an automatic decoy database search. The peptide searching parameters included a precursor mass tolerance of 15 ppm, a fragment ion mass tolerance of 20 mmu, a tolerance of one missed cleavage of trypsin, the methylthiol group of cysteine as the fixed modification, and Gln→pyro-Glu (N-term Q), Oxidation (M), iTRAQ 8 plex (K), iTRAQ 8 plex (N-term), and iTRAQ 8 plex (Y) as variable modifications. After database searching, the DAT files were loaded into the scaffold_3.6.4 software (Proteome Software, Inc.) for protein identification and quantification. The proteins with at least two unique peptides and a false discovery rate (FDR) less than 0.01 were qualified for further quantification analysis. At each time point, the sample from WT mice was set as the quantification reference. The fold changes in protein abundance were defined as the median ratios of tag intensities from all significantly matched spectra between Apc^Min/+^ and WT samples. Because IPI database was shut down, the accession numbers of UniProt (unreviewed) for all the proteins searched in IPI database were provided, and protein names were substituted based on UniProt database (Supplementary Table 2).

### Preparation of TIF/serum tryptic peptides for LC-MRM-MS

The ProteoMiner protein enrichment kit (BIO-RAD, Hercules, CA) was applied to enrich the serum proteins with low abundance according to the manufacturer's instructions. A total of 30 μg proteins from six individual TIF or six enriched serum samples were separated by 12% SDS-PAGE, and the gel bands with molecular weights of 25-30 kDa were dissected into small and uniform pieces, which were then subjected to in-gel tryptic digestion [40]. Subsequently, the lyophilized peptides were dissolved in 2% acetonitrile with 0.1% aqueous formic acid, and each sample was spiked with β-Galactosidase (BG) peptides at a final concentration of 2 fmol/μL as an internal standard for quality control and the normalization of MRM signals.

### Candidate verification using LC-MRM-MS

Approximately 5 μg of in-gel digested peptides from a pooled TIF or serum sample were analyzed on a TripleTOF 5600 or 6600 System (AB SCIEX, Concord, NH) equipped with a nano LC system (Shimadzu, Kyoto, Japan) to identify target peptides with significant MS/MS signals corresponding to the protein candidates. The data were then searched with ProteinPilot (AB SCIEX) against the IPI mouse or IPI human database, and the search results were further imported to Skyline v2.1 (MacCoss Lab) to establish the MRM transition list. Finally, the selected transitions were adopted to survey the protein digests from the individual TIF and serum samples. All MRM samples were analyzed in triplicate using a QTRAP 6500 MS (AB SCIEX, Framingham, MA) equipped with an ekspert nano LC 425 system (Eksigent, Silicon Valley, CA). The mobile phases consisted of solvent A (2% acetonitrile with 0.1% aqueous formic acid) and solvent B (100% acetonitrile with 0.1% formic acid). The peptides were separated on an eksigent column (75 μmx15 cm, 3 μm particles, 120 Å aperture, Eksigent, Silicon Valley, CA) at 300 nL/min with a gradient of 5-40% solvent B for 29 min and 40%-80% solvent B for 15 min. The MS parameters were set as follows: ion spray voltage at 2,300 V, curtain gas at 35.0, ion source gas1 at 25.0, ion source gas2 at 0.0, collision gas at high, interface heater temperature at 150.0°C, entrance potential at 10.0, and Q1 and Q3 at unit resolution. In the MRM mode, the declustering potential and collision energy were calculated by skyline v2.1. To ensure that the peptides detected by MRM could represent the target proteins, the corresponding peptides were chemically synthesized (GL Biochem, Shanghai, China) and further analyzed by MRM assays to validate their retention time and overlays. The human homologues of the six mouse serine proteases were determined as the proteins with the highest scores by aligning mouse protein sequences in human database.

### MRM data processing

The MRM raw data were processed with skyline v2.1. To ensure correct peak detection and integration, the data were manually inspected to further filter the peptides. The median values for the MRM peak areas of the target peptides in the TIFs/sera of one 13-week-old Apc^Min/+^ mouse, the pooled TIF/serum from 22-week WT mice or one control healthy serum sample were set as normalization references. The intensities of all precursors were adjusted with those of spiked BG peptides in the corresponding samples, and the relative protein abundance of each target protein with two unique peptides was determined by averaging the two corresponding peptides. The Mann-Whitney U test was employed to evaluate the significance of differences. ROC curves were generated using the SPSS version 17.0 statistical package. *p*<0.05 was considered to indicate significant differences. To obtain the diagnostic utility of a combination, the predicted probability was generated with a logistic regression using SPSS, and this value was then used as a surrogate marker to construct an ROC curve. The AUC was used to evaluate the accuracy of a marker. The sensitivity and specificity were computed according to Youden's index.

### TMA analysis

TMAs containing 80 paired samples from colon cancer tissues and their adjacent tissues were purchased from Shanghai Outdo Biotech Co. (Table 2) and analyzed by immunohistochemistry according to Wang's protocol [41] with mouse anti-CELA1 (1:200, Abcam, Hong Kong, China) or rabbit anti-CTRL antibody (1:200, Proteintech, Chicago, IL). The correlation between CELA1 or CTRL and the clinical pathological traits of colon cancer were calculated by the chi-square test using SPSS.

## SUPPLEMENTARY FIGURES AND TABLES




