# Supplementary material for: The levels of serine proteases in colon tissue interstitial fluid and serum serve as an indicator of colorectal cancer progression
Source: Oncotarget. 2016 Apr 11;7(22):32592–606. doi: 10.18632/oncotarget.8693 (PMC5078036; doi:10.18632/oncotarget.8693)
Supplement: Supplementary file 1 [file oncotarget-07-32592-s001.pdf]

The levels of serine proteases in colon tissue interstitial fluid and serum serve as an indicator of colorectal cancer progression

SUPPLEMENTARY FIGURES AND TABLES

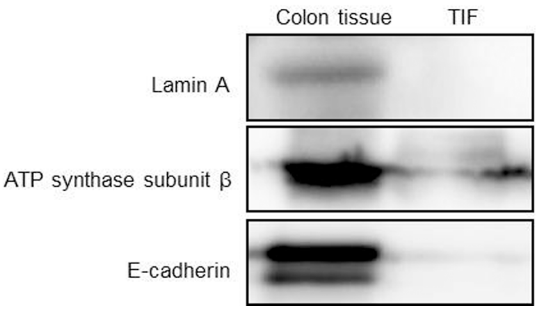

Supplementary Figure S1: Quality evaluation of pooled mouse colon TIF using Western blotting. Lamin A, ATP synthase subunit β, and E-cadherin were used as nuclear, mitochondrial, and plasma membrane markers, respectively. The TIF sample was mixed from 12 $Apc^{Min/+}$ mice, three at each time point.

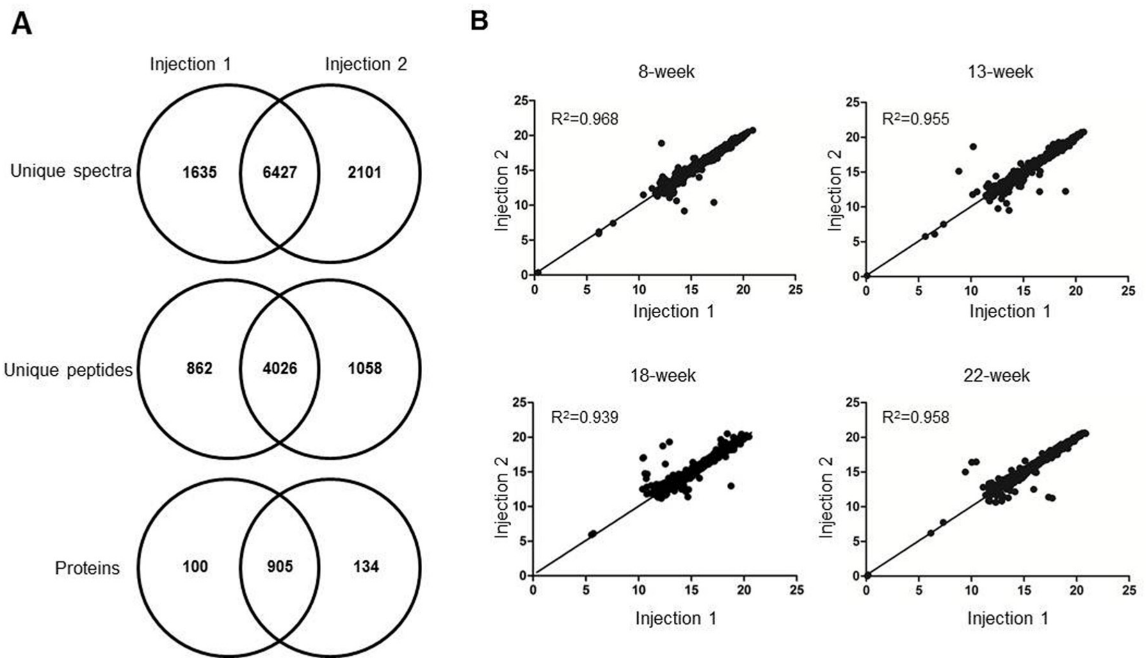

Supplementary Figure S2: The qualitative and quantitative reproducibility of technical duplicated LC-MS/MS data. **A.** Overlaps of unique spectra, peptides and proteins identified in duplicate. **B.** Correlation analysis of normalized tag intensities in duplicate using the Pearson method. The square of the correlation coefficient (R^2) is listed.

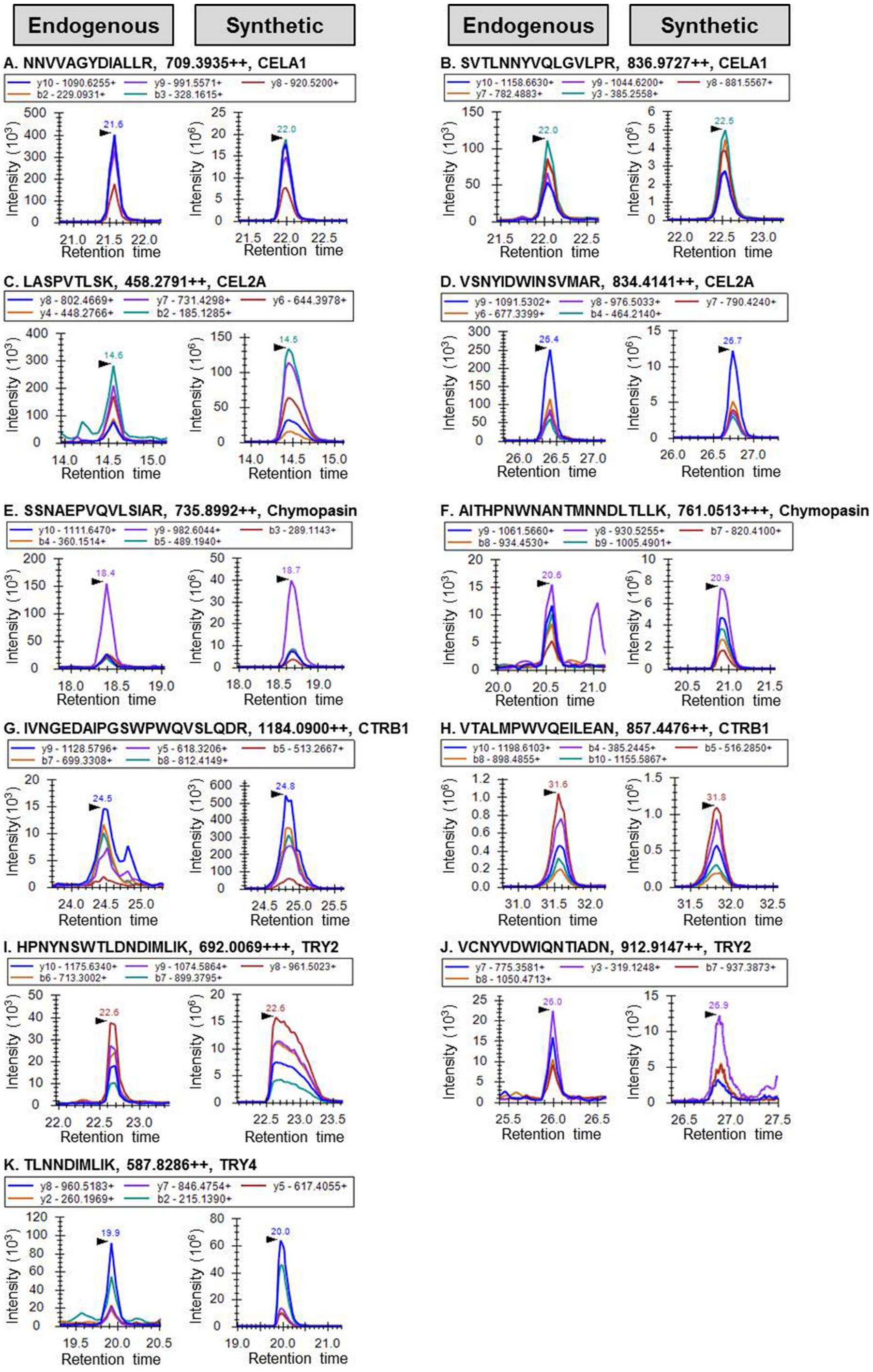

Supplementary Figure S3: Validation of the MRM signals in mouse TIFs and sera. For each peptide, the left panel shows the endogenous peptide detected in mouse samples, and the right panel shows the corresponding synthetic peptide as a validation standard to determine retention time and overlays.

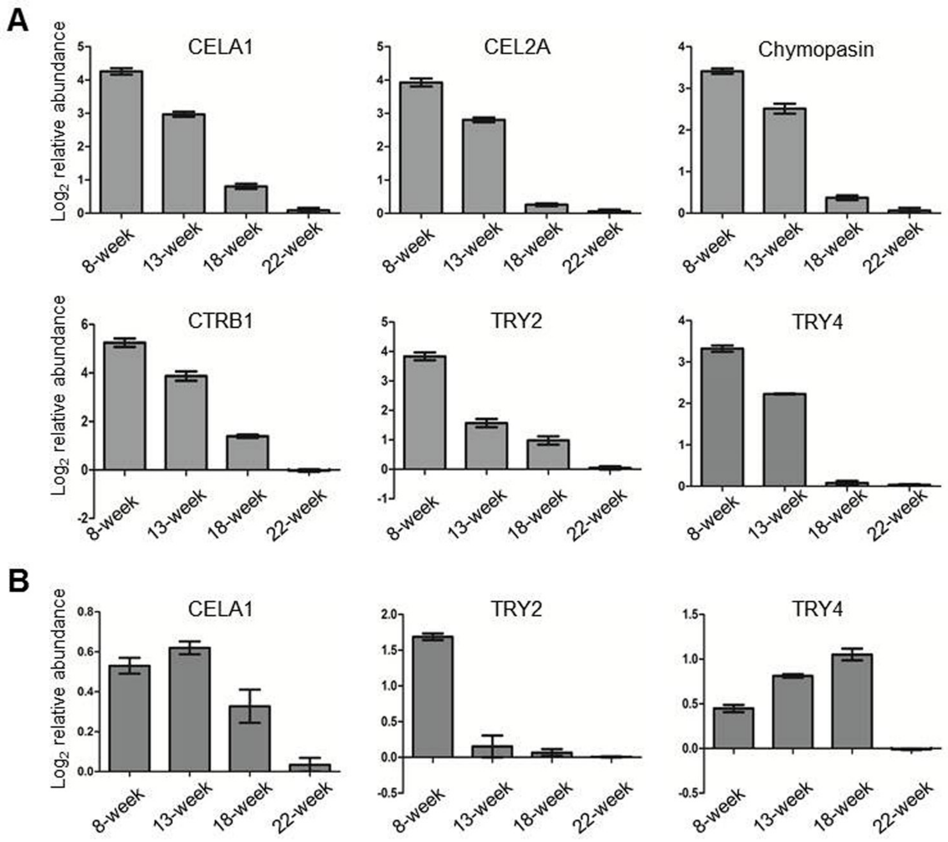

Supplementary Figure S4: The relative protein levels of the detectable serine proteases in pooled TIF (A) and serum (B) samples from WT mice (n=3/time point) measured using MRM. The protein peak areas of 22-week-old TIF and serum samples were used as the references.

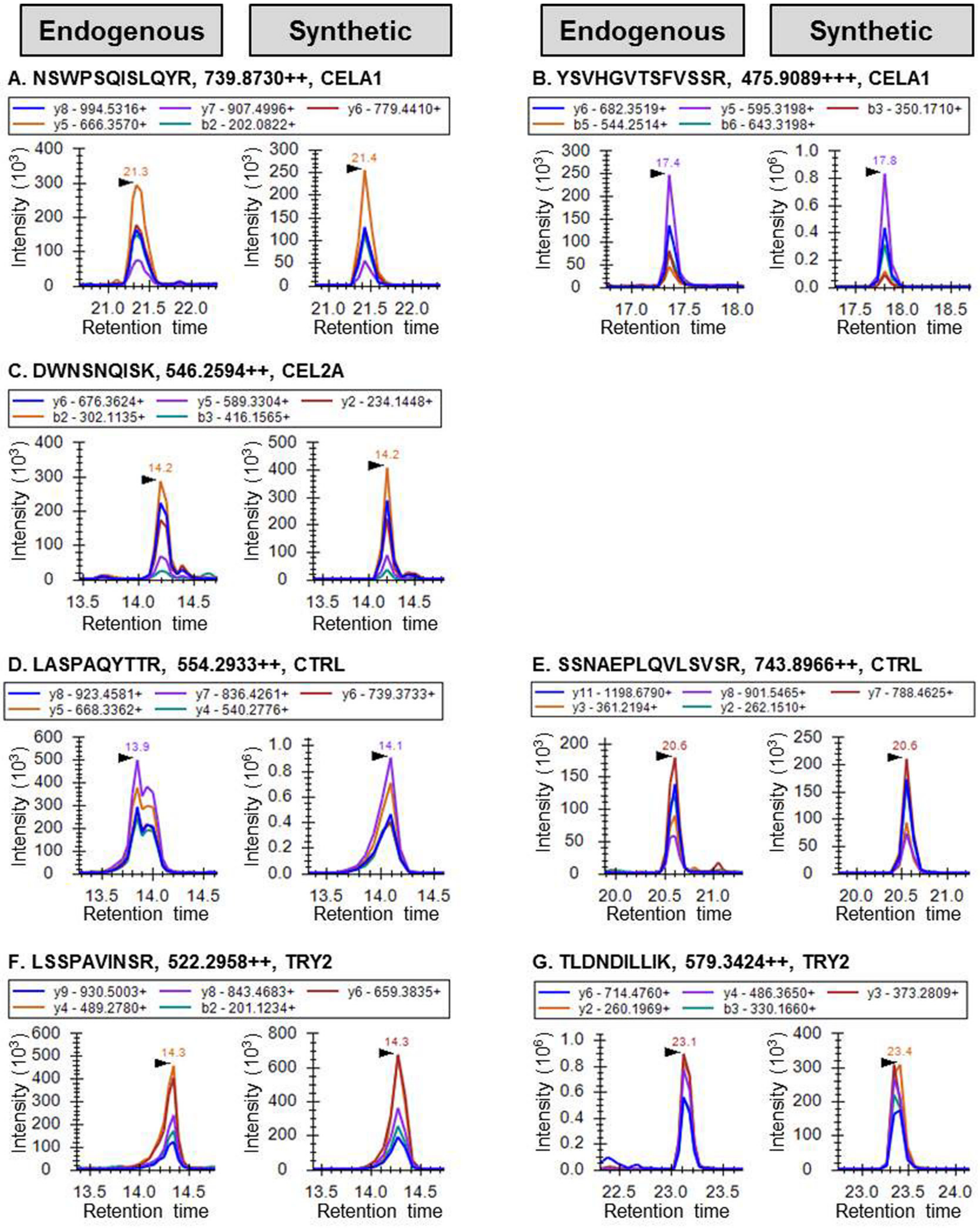

Supplementary Figure S5: Validation of the MRM signals in human sera. For each peptide, the left panel shows the endogenous peptide detected in serum, and the right panel shows the corresponding synthetic peptide as a validation standard to determine retention time and overlays.

Supplementary Table S1: Number and size of colorectal tumors in individual mice at four time points

Age of mice	Mouse ID	Number of tumors	Average number (Mean±SE)	Size of tumors (mm)	Average size (Mean±SE)
	1	0		0	
	2	0		0	
	3	0		0	
	4	0		0	
8-week	5	0	0	0	0
	6	0		0	
	7	0		0	
	8	0		0	
	9	0		0	
	1	1		1.3	
	2	1		1.8	
	3	1		2	
13-week	4	1	1.14±0.34	1.6	1.54±0.11
	5	3		1.1, 1.6, 1.2	
	6	1		1.7	
	7	0		0	
	1	1		3.1	
	2	2		3.5, 2.4	
18-week	3	2	1.50±0.22	2.8, 3.0	2.98±0.12
	4	2		2.6, 3.4	
	5	1		3.2	
	6	1		2.8	
	1	3		4.7, 3.9, 5.0	
	2	3		4.2, 4.5, 4.8	
	3	2		4.0, 4.3	
	4	3		5.3, 6.1, 4.3	
22-week	5	4	2.50±0.33	6.2, 4.5, 4.7, 3.9	4.64±0.16
	6	2		4.2, 4.0	
	7	2		3.8, 5.8	
	8	1		4.5	

Supplementary Table S2: Information for 1174 identified proteins

See Supplementary File 1

Supplementary Table S3: Peptide parameters of the six serine proteases for MRM in mouse TIFs and sera

Protein name	Unique peptide	Charge	Q1	Q3	Fragment
CELA1	NNVVAGYDIALLR	2+	709.3935	1090.6255, 991.5571, 920.5200, 229.0931, 328.1615	y10,y9,y8,b2,b3
	SVTLNNYVQLGVLPR	2+	836.9727	1158.6630, 1044.6200, 881.5567, 782.4883, 385.2558	y10,y9,y8,y7,y3
CEL2A	LASPVTLSK	2+	458.2791	802.4669, 731.4298, 644.3978, 448.2766, 185.1285	y8,y7,y6,y4,b2
	VSNYIDWINSVMAR	2+	834.4141	1091.5302, 976.5033, 790.4240, 677.3399, 464.2140	y9,y8,y7,y6,b4
Chymopasin	SSNAEPVQVLSIAR	2+	735.8992	1111.6470, 982.6044, 289.1143, 360.1514, 489.1940	y10,y9,b3,b4,b5
	AITHPNWNANTMNNDLTLLK	3+	761.0513	1061.5660, 930.5255, 820.4100, 934.4530, 1005.4901	y9,y8,b7,b8,b9
CTRB1	IVNGEDAIPGSWPWQVSLQDR	2+	1184.09	1128.5796, 618.3206, 513.2667, 699.3308, 812.4149	y9,y5,b5,b7,b8
	VTALMPWVQEILEAN	2+	857.4476	1198.6103, 385.2445, 516.2850, 898.4855, 1155.5867	y10,b4,b5,b8,b10
TRY2	HPNYNSWTLDNDIMLIK	3+	692.0069	1175.6340, 1074.5864, 961.5023, 713.3002, 899.3795	y10,y9,y8,b6,b7
	VCNYVDWIQNTIADN	2+	912.9147	775.3581, 319.1248, 937.3873, 1050.4713	y7,y3,b7,b8
TRY4	TLNNDIMLIK	2+	587.8286	960.5183, 846.4754, 617.4055, 260.1969, 215.1390	y8,y7,y5,y2,b2

Supplementary Table S4: Peptide parameters of the four serine proteases for MRM in human sera

Protein name	Unique peptide	Charge	Q1	Q3	Fragment
CELA1	NSWPSQISLQYR	2+	739.873	994.5316, 907.4996, 779.4410, 666.3570, 202.0822	y8, y7, y6, y5, b2
	YSVHGVTSFVSSR	3+	475.9089	682.3519, 595.3198, 350.1710, 544.2514, 643.3198	y6, y5, b3, b5, b6
CEL2A	DWNSNQISK	2+	546.2594	676.3624, 589.3304, 234.1448, 302.1135, 416.1565	y6, y5, y2, b2, b3
CTRL	SSNAEPLQVLSVSR	2+	743.8966	1198.6790, 901.5465, 788.4625, 361.2194, 262.1510	y11, y8, y7, y3, y2
	LASPAQYTTR	2+	554.2933	923.4581, 836.4261, 739.3733, 668.3362, 540.2776	y8.,y7, y6, y5, y4
TRY2	TLDNDILLIK	2+	579.3424	714.4760, 486.3650, 373.2809, 260.1969, 330.1660	y6, y4, y3, y2, b3
	LSSPAVINSR	2+	522.2958	930.5003, 843.4683, 659.3835, 489.2780, 201.1234	y9, y8, y6, y4, b2
